# Supplementary material for: Non-canonical NF-κB signaling limits the tolerogenic β-catenin-Raldh2 axis in gut dendritic cells to exacerbate intestinal pathologies
Source: EMBO J. 2024 Jul 25;43(18):3895–915. doi: 10.1038/s44318-024-00182-6 (PMC11405688; doi:10.1038/s44318-024-00182-6)
Supplement: Supplementary file 1 — Appendix [file 44318_2024_182_MOESM1_ESM.pdf]

Appendix

Non-canonical NF-κB signaling limits the tolerogenic β-catenin-Raldh2 axis in gut dendritic cells to exacerbate intestinal pathologies

Alvina Deka[1,*], Naveen Kumar[1,*], Swapnava Basu[1], Meenakshi Chawla[1], Namrata Bhattacharya[2,3], Sk Asif Ali[1], Bhawna[1], Upasna Madan[4], Shakti Kumar[4], Bhabatosh Das[4], Debarka Sengupta[2], Amit Awasthi[4], Soumen Basak[1, ✉]

[1]Systems Immunology Laboratory, National Institute of Immunology, Aruna Asaf Ali Marg, New Delhi-110067, India

[2]Indraprastha Institute of Information Technology, Delhi, India

[3]Australian Prostate Cancer Research Centre—Queensland, Institute of Health and Biomedical Innovation, School of Biomedical Sciences, Queensland University of Technology (QUT), Brisbane, Queensland, Australia

[4]Translational Health Science and Technology Institute, Faridabad, India

* these authors have contributed equally to the work

✉ Correspondence should be addressed to S.B.;
e-mail: sobasak@nii.ac.in; tel: (91) (11) 26703853; fax: (91) (11) 2674262

Table of contents:

Appendix Figure S1: Studying DSS-induced experimental colitis in mice. Page - 2-3
Appendix Figure S2: Studying WT and non-canonical NF-κB-deficient BMDCs. Page - 4
Appendix Figure S3: Investigating colonic lamina propria immune cells in mice. Page - 5
Appendix Figure S4: Investigating crosstalk between b-catenin and non-canonical NF-kB signaling in DCs. Page - 6
Appendix Figure S5: Probing the non-canonical NF-κB signaling in intestinal MNPs from IBD patients Page - 6
Appendix Table S1: Prototypic gene signatures for cell types/subsets Page - 7

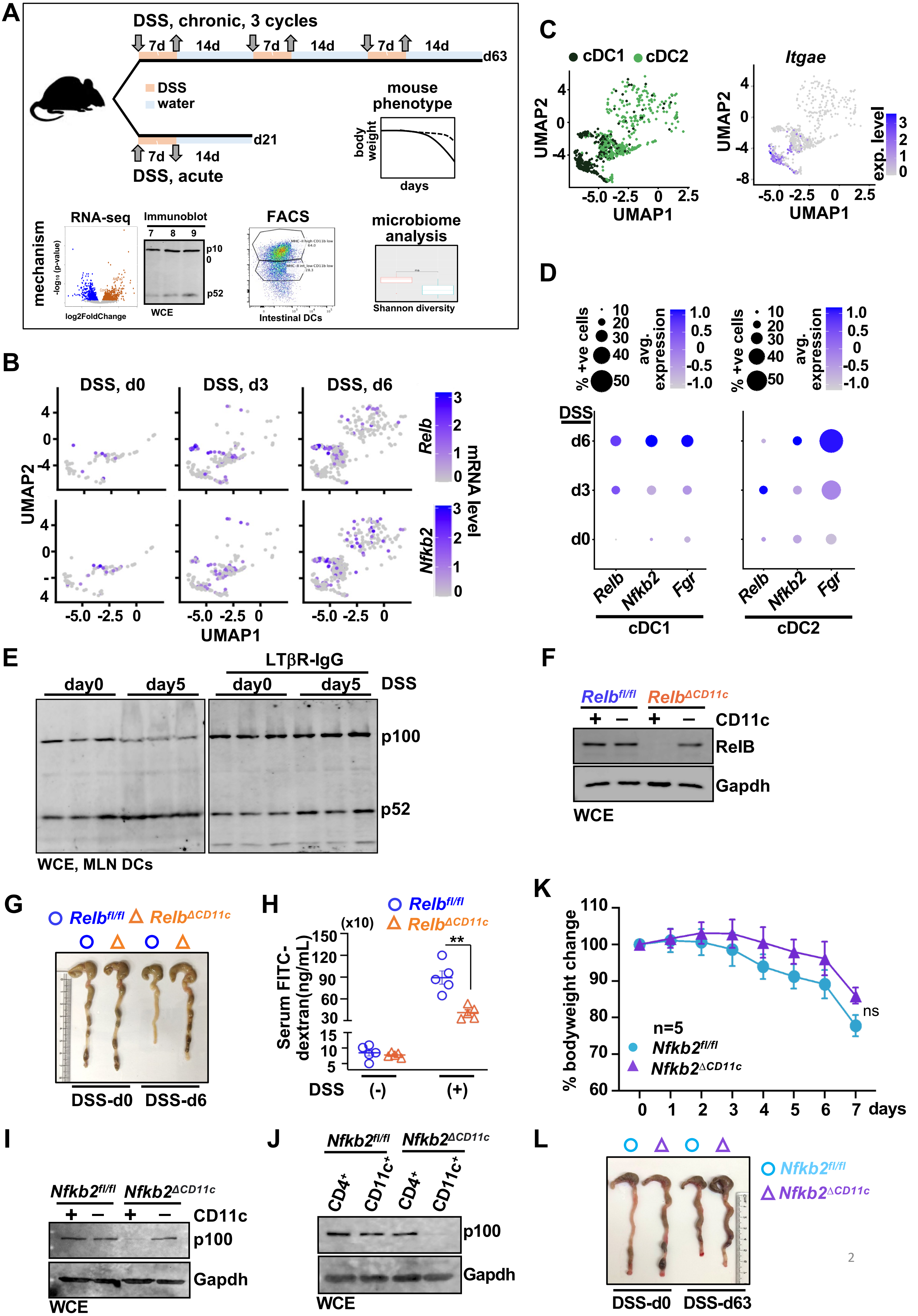
A
DSS, chronic, 3 cycles
7d 14d 7d 14d 7d 14d d63
DSS
water
d21
7d 14d
DSS, acute
mouse phenotype
body weight
days
mechanism
RNA-seq
log2FoldChange
Immunoblot
7 8 9
p100
p52
WCE
FACS
Intestinal DCs
microbiome analysis
Shannon diversity
B
DSS, d0
DSS, d3
DSS, d6
UMAP2
UMAP1
Relb
Nfkb2
mRNA level
C
cDC1 cDC2
Itgae
UMAP2
UMAP1
exp. level
D
% +ve cells
avg. expression
DSS
d6
d3
d0
Relb Nfkb2 Fgr
cDC1
cDC2
E
LTβR-IgG
day0 day5 day0 day5 DSS
p100
p52
WCE, MLN DCs
F
Relb^fl/fl Relb^ΔCD11c
CD11c
RelB
Gapdh
WCE
G
Relb^fl/fl Relb^ΔCD11c
DSS-d0 DSS-d6
H
Relb^fl/fl
Relb^ΔCD11c
(x10)
Serum FITC-dextran(ng/mL)
**
DSS (-) (+)
K
% bodyweight change
n=5
Nfkb2^fl/fl
Nfkb2^ΔCD11c
ns
days
I
Nfkb2^fl/fl Nfkb2^ΔCD11c
CD11c
p100
Gapdh
WCE
J
Nfkb2^fl/fl Nfkb2^ΔCD11c
CD4+ CD11c+ CD4+ CD11c+
p100
Gapdh
WCE
L
Nfkb2^fl/fl
Nfkb2^ΔCD11c
DSS-d0 DSS-d63

Appendix Figure S1: Studying DSS-induced experimental colitis in mice. (A) Experimental schema for DSS-induced acute and chronic colitis in mice. Immunoblot from Figure 2A has been re-used here for representation purpose. **(B)** UMAP depicting the expression of Relb and Nfkb2 mRNAs in a time course in intestinal DCs from WT mice subjected to DSS-induced acute colitis **(C)** Feature plot depicting the DC-subsets (left panel) and CD103 expression (right) in the DC cluster indicated in Fig1A. The list of genes used for distinguishing cDC1 and cDC2 subpopulations has been provided in supplementary Table 1. **(D)** Dotplot revealing the expression of indicated genes in cDC1 (left) and cDC2 (right). Publicly available mouse single-cell RNA-seq data was analysed (GSE148794). **(E)** Immunoblot showing abundance of p52/p100 in MLN-DCs sorted from untreated mice or those administered with 1.5% DSS in drinking water for 5 days. Also, the mice were treated with indicated antibodies on day -1, 2, 4 of the DSS treatment. **(F)** Immunoblots revealing the abundance of RelB in $CD11c^{+}$ or $CD11c^{-}$ splenocytes from *Relb*$^{fl/fl}$ and *Relb*$^{\Delta CD11c}$ mice. **(G)** Representative colon images from *Relb*$^{fl/fl}$ and *Relb*$^{\Delta CD11c}$ mice subjected to 1.5% acute DSS treatment. **(H)** Dot plot revealing the serum concentration of FITC-dextran in *Relb*$^{fl/fl}$ and *Relb*$^{\Delta CD11c}$ mice left untreated or subjected to acute DSS treatment; FITC-dextran was gavaged orally 6h prior to serum collection **(I), (J)** Immunoblot showing p100/*Nfkb2* levels in $CD11c^{+}$ and $CD11c^{-}$ (H) or CD4+ and CD4- (I) splenocytes from *Nfkb2*$^{fl/fl}$ and *Nfkb2*$^{\Delta CD11c}$ mice. **(K)** In the acute colitis regime, *Nfkb2*$^{fl/fl}$ and *Nfkb2*$^{\Delta CD11c}$ mice were treated with 1.5% DSS and evaluated for body weight changes for 7 days **(L)** Representative colon images from *Nfkb2*$^{fl/fl}$ and *Nfkb2*$^{\Delta CD11c}$ mice subjected to chronic-DSS treatment. Data represent mean $\pm$ SEM. For statistical analysis, two-tailed Student's t-test was performed. ns, not significant.

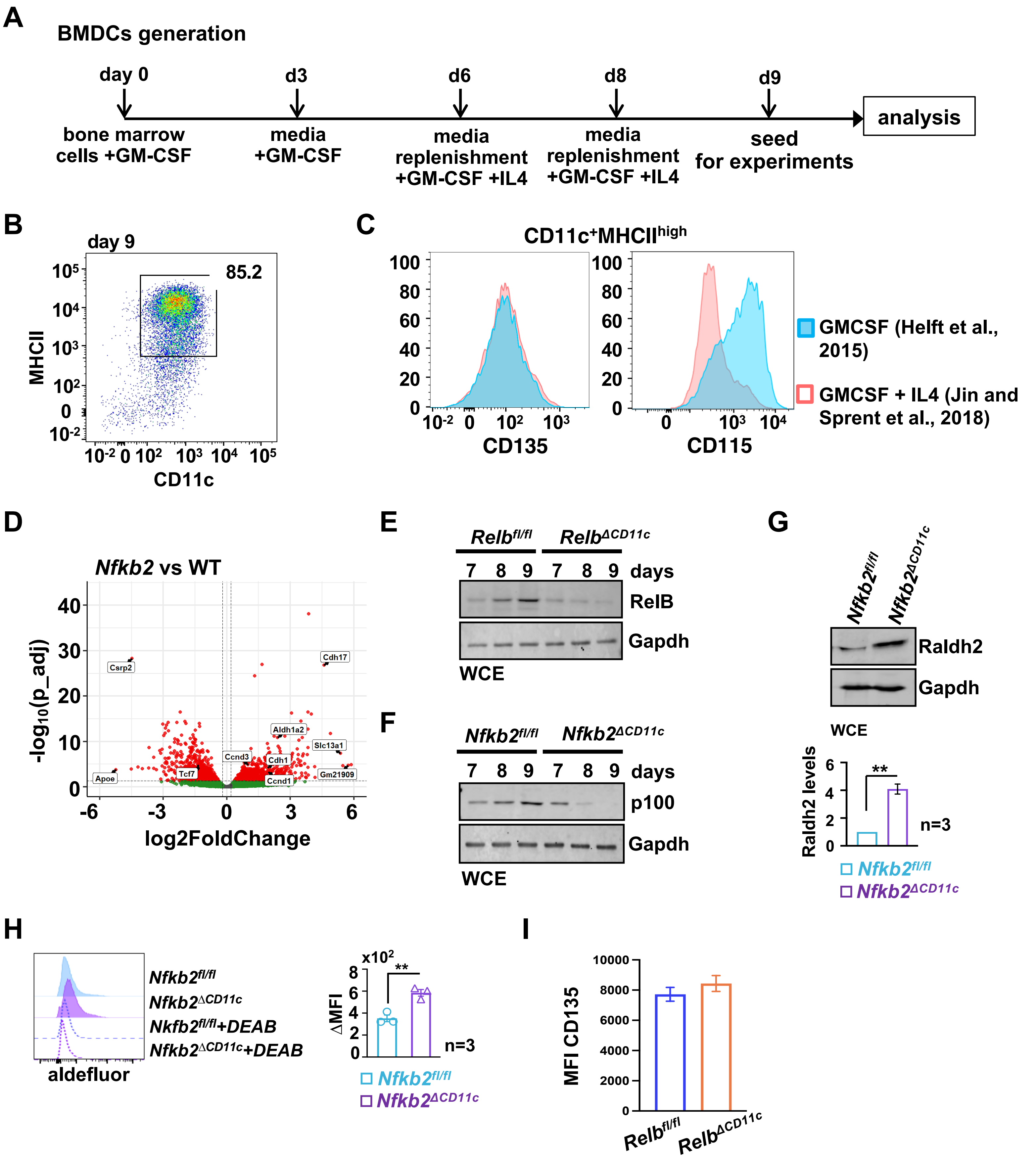

Appendix Figure S2: Studying WT and non-canonical NF-κB-deficient BMDCs. (A) Schema depicting generation BMDCs *ex vivo*. **(B)** Representative FACS plot showing the percentage of $CD11c^{+}MHCII^{hi/int}$ cells generated on day 9 in BMDC-differentiating culture *ex vivo*. Bone marrow cells from WT C57/BL6 mice were used. **(C)** Histogram comparing $CD11c+MHCII^{high}$ BMDCs generated using either GM-CSF alone following the protocol published by Helft et al., (2015) or a cocktail of GM-CSF+IL4 following the protocol published by Jin and Sprent et al., (2018) for the surface expression of the DC marker CD135 or the macrophage marker CD115. **(D)** Volcano plot comparing WT and $Nfkb2^{-/-}$ BMDCs for the global gene expression. **(E), (F)** Immunoblot analyses comparing BMDCs from $Relb^{fl/fl}$ and $Relb^{\Delta CD11c}$ **(E)** or $Nfkb2^{fl/fl}$ and $Nfkb2^{\Delta CD11c}$ **(F)** mice for the expression of RelB **(E)** or p100 **(F)**, respectively. **(G)** Representative immunoblot revealing the abundance of Raldh2 in $Nfkb2^{fl/fl}$ and $Nkfb2^{\Delta CD11c}$ BMDCs. Barplot below represent quantified band intensities. **(H)** Histograms (left) showing Raldh enzymatic activity measured by Aldefluor assay in $Nfkb2^{fl/fl}$ and $Nkfb2^{\Delta CD11c}$ BMDCs. **(I)** Bargram comparing $Relb^{fl/fl}$ and $Relb^{\Delta CD11c}$ BMDCs for the MFI of surface expressions of the DC marker CD135. Data from independent experiments are presented in a barplot (right). Data represent mean ± SEM. For statistical analysis, two-tailed Student's t-test was performed. *$P < 0.05$; **$P < 0.01$; ***$P < 0.001$; ns, not significant.

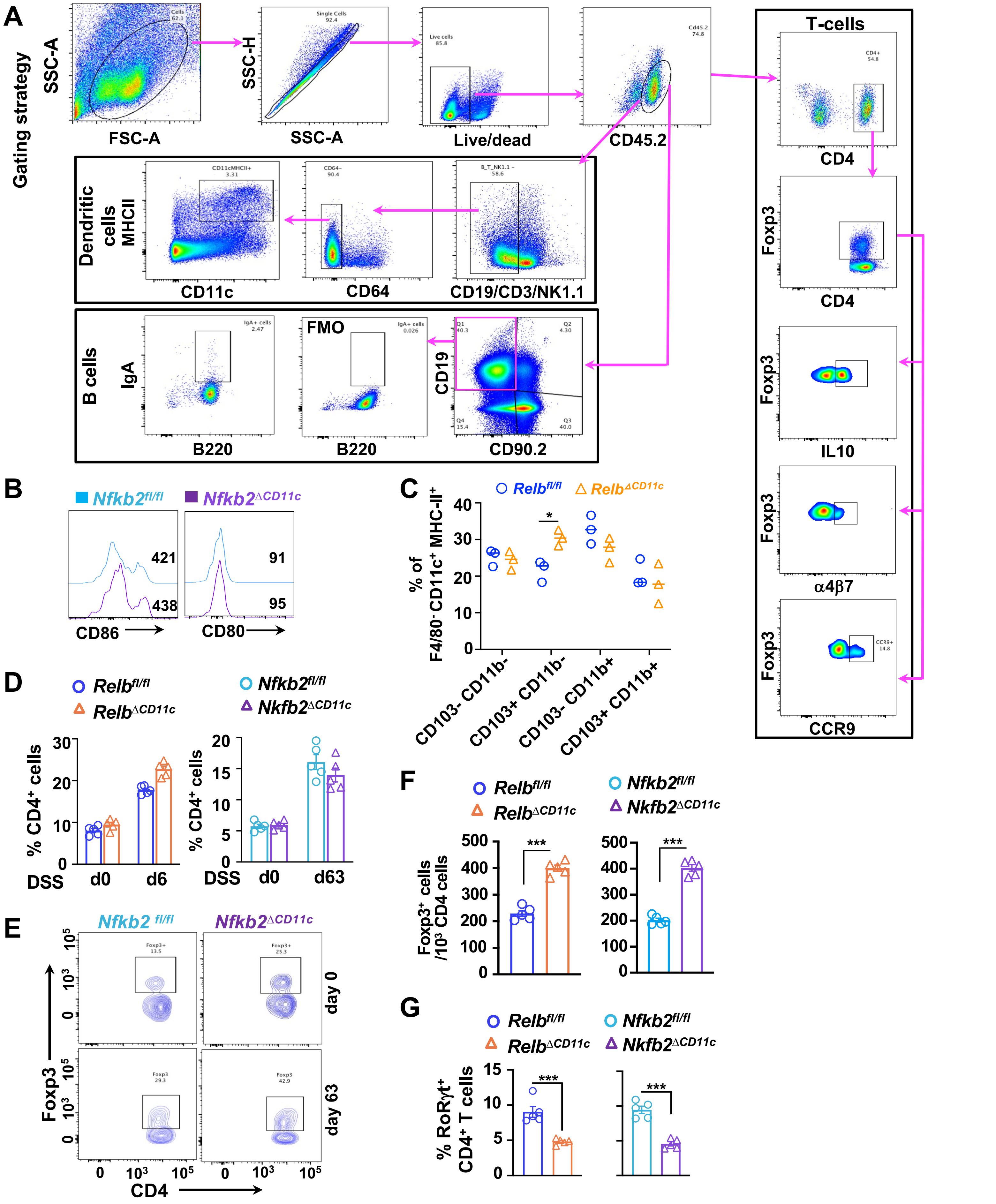

Appendix Figure S3: Investigating colonic lamina propria immune cells in mice. (A) Representative FACS plots showing the gating strategy for analyzing the frequencies of dendritic cells, T cell subsets and B-cells in the lamina propria. Fluorescence minus one(FMO) was used as a gating control for gating IgA+ cells. A FACS plot from Figure 4A has been re-used here for representation purpose. **(B)** Histograms comparing CD80 and CD86 levels in MLN or LP DCs from *Nfkb2*$^{fl/fl}$ and *Nfkb2*$^{\Delta CD11c}$ mice. **(C)** Scatter plot depicting the frequency of indicated dendritic cell subsets in MLN from *Relb*$^{fl/fl}$ and *Relb*$^{\Delta CD11c}$ mice. **(D)** Barplot revealing the frequency of CD4+ T cells as a percentage of CD45.2+ cells in the lamina propria of the indicated mice mice (n=5). **(E)** Flow cytometry analyses showing the frequency of FoxP3+ Tregs in LP or MLNs of Nfkb2$^{fl/fl}$ and Nfkb2$^{\Delta CD11c}$ mice, untreated or subjected to chronic DSS treatment. **(F).** Barplot indicating the number of Foxp3+ T-cells/10^3 CD4+ cells **(E)** in the lamina propria of the indicated mice (n=5). **(G)** Barplot with the frequency of RoRγt+ cells as a percentage of CD4 T cells in the LP of indicated mice. Data represent mean ± SEM. For statistical analysis, two-tailed Student's t-test was performed. *P < 0.05; **P < 0.01; ***P <

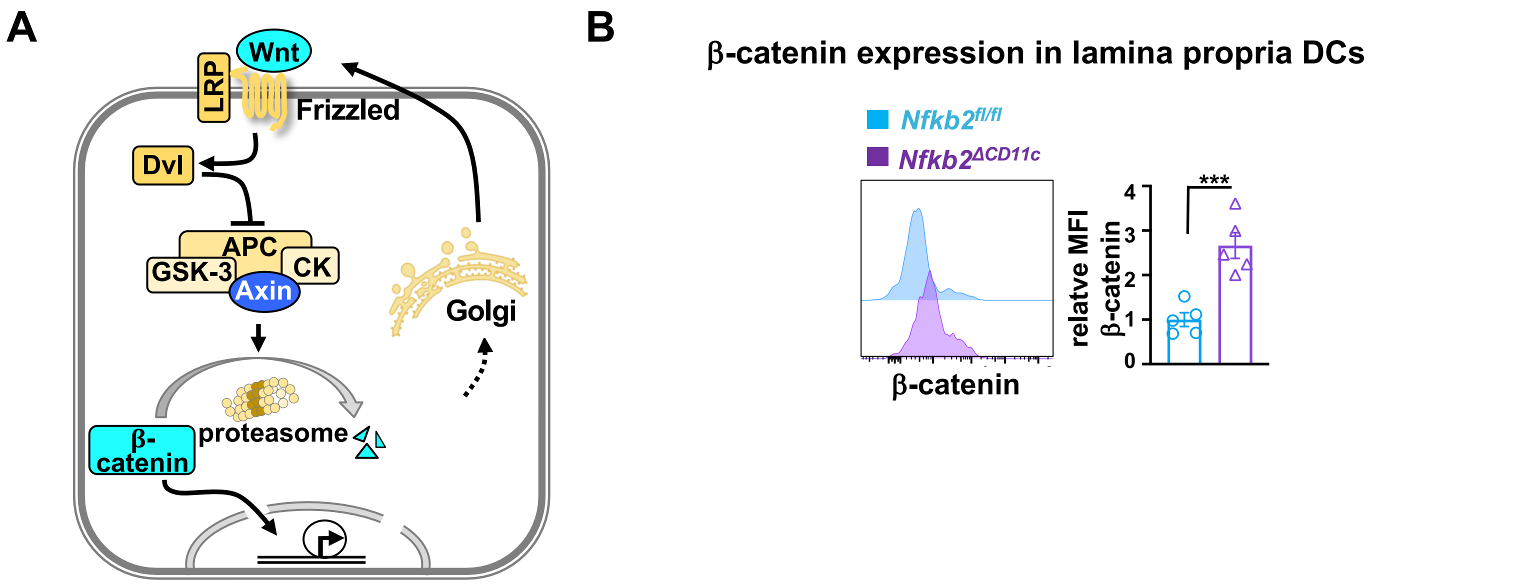

Appendix Figure S4: Investigating crosstalk between β-catenin and non-canonical NF-κB signaling in DCs. (A) A cartoon describing β-catenin-regulatory mechanisms. **(B)** Representative histogram comparing MLN DCs from *Nfkb2*$^{fl/fl}$ and *Nfkb2*$^{\Delta CD11c}$ mice for the expression of β-catenin.Corresponding quantified data showing relative MFI for β-catenin expression has also been presented in a barplot (right). Data represent mean $\pm$ SEM. For statistical analysis, two-tailed Student's t-test was performed. *P < 0.05; **P < 0.01; ***P < 0.001.

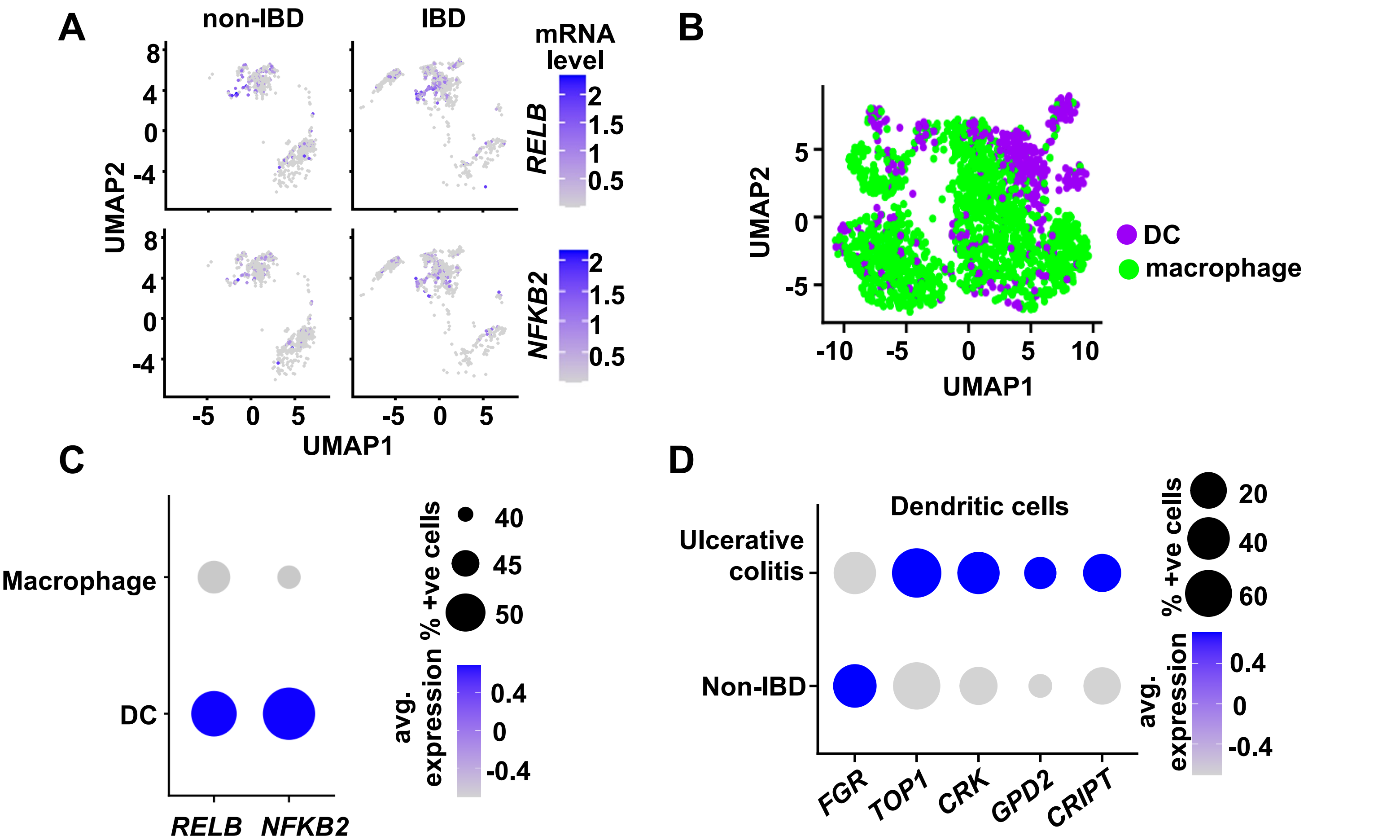

Appendix Figure S5: Probing the non-canonical NF-κB signaling in intestinal MNPs from IBD patients. (A) UMAP comparing IBD patients and non-IBD subjects for the level of RELB and NFKB2 mRNAs in intestinal DCs; the intensity of dot color denotes the abundance of corresponding mRNAs in each cell. Single-cell RNA-seq data available at Single Cell Portal was used for analyses (Ref: SCP 259). **(B, C, D)** Briefly, an additional colonic single-cell RNA-seq dataset for IBD patients available at the NCBI database was also used for analyses. (Ref: GSE162335) **(B)** UMAP depicting the DC and macrophage cell population among mononuclear phagocytes in the gut lamina propria from the gut biopsies of ulcerative colitis patients and non-IBD individuals. **(C)** Dot plot comparing DCs and macrophages from the lamina propria of human subjects for the expression of *RELB* and *NFKB2* mRNAs. **(D)** Dot plot revealing the expression of indicated RelB-important mRNAs in DCs from gut biopsies of ulcerative colitis patients and control subjects.

Appendix Table S1- Prototypic gene signatures for cell types/subsets

Cell type/subset	Signature genes
Dendritic cells (adopted from Xu *et al.*, 2019[9])	*Cd74, H2ab1, H2eb1, H2aa, Tmsb4x, Cst3, Cd52, H2dma, Psap, Crip1, Sh3bgrl3, Tyrobp, Lsp1, Fcer1g, Coro1a, Mpeg1, Irf8, Plbd1, Gpx1, Lgals3, Srgn, Ctss, Alox5ap, Ifi30, Gm2a, Xcr1, Clec9a, Itgax, Ccr2, Clec7a, Cd83, Cd86, Batf3, Ccr7, Zbtb46, Bcl11a, Flt3, Dpp4, Cd8a, Cd14, Csf1r, Cx3cr1, Fcgr3, Fcgr1, Cd207, Cd209a, Itgam, Sirpa, Cd4, Irf4, Tbx21, Mgl2, Esam, Dtx1, Rbpj, Siglech, Irf7, Tcf4, Bst2, Il23a, Cxcr3, Ccr6*
Macrophages (adopted from Xu *et al.*, 2019[9])	*Ccl8, Apoe, C1qb, C1qc, Lyz2, C1qa, Ctsb, Selenop, Itm2b, Fth1, H2-d1, Tmsb4x, Tyrobp, Ctss, Pf4, B2m, Ftl1, Lgmn, Grn, Fcgr3, Csf1r, Cst3, Laptm5, Mrc1, Wfdc17, F13a1, Serinc3, Fcer1g, Ctsc, Cd74, Clec4f, Cd5l, Vsig4, Fcna, Psap, Mafb, Cd68, Cd163, Cd163l1, Mertk, Cd209, Stab1, Slco2b1,mmp12, Mmp14, F480, Itgam, Cx3cr1, Fcgr1, Adgre1, Cd64*
Type-l Classical DC (cDC1) (adopted from Brown *et al.,* 2019)	*Xcr1, Batf3, Irf8, Cd8a, Itgae, Flt3, Clec9a, Ly75*
Type-II Classical DC (cDC2) (adopted from Brown *et al.*, 2019)	*Itgam, Sirpa, Irf4, Csf1r, Zbtb46, Marco, Clec4*
