# Supplementary figures and images for: Non-canonical NF-κB signaling limits the tolerogenic β-catenin-Raldh2 axis in gut dendritic cells to exacerbate intestinal pathologies
Source: EMBO J. 2024 Jul 25;43(18):3895–915. doi: 10.1038/s44318-024-00182-6 (PMC11405688; doi:10.1038/s44318-024-00182-6)

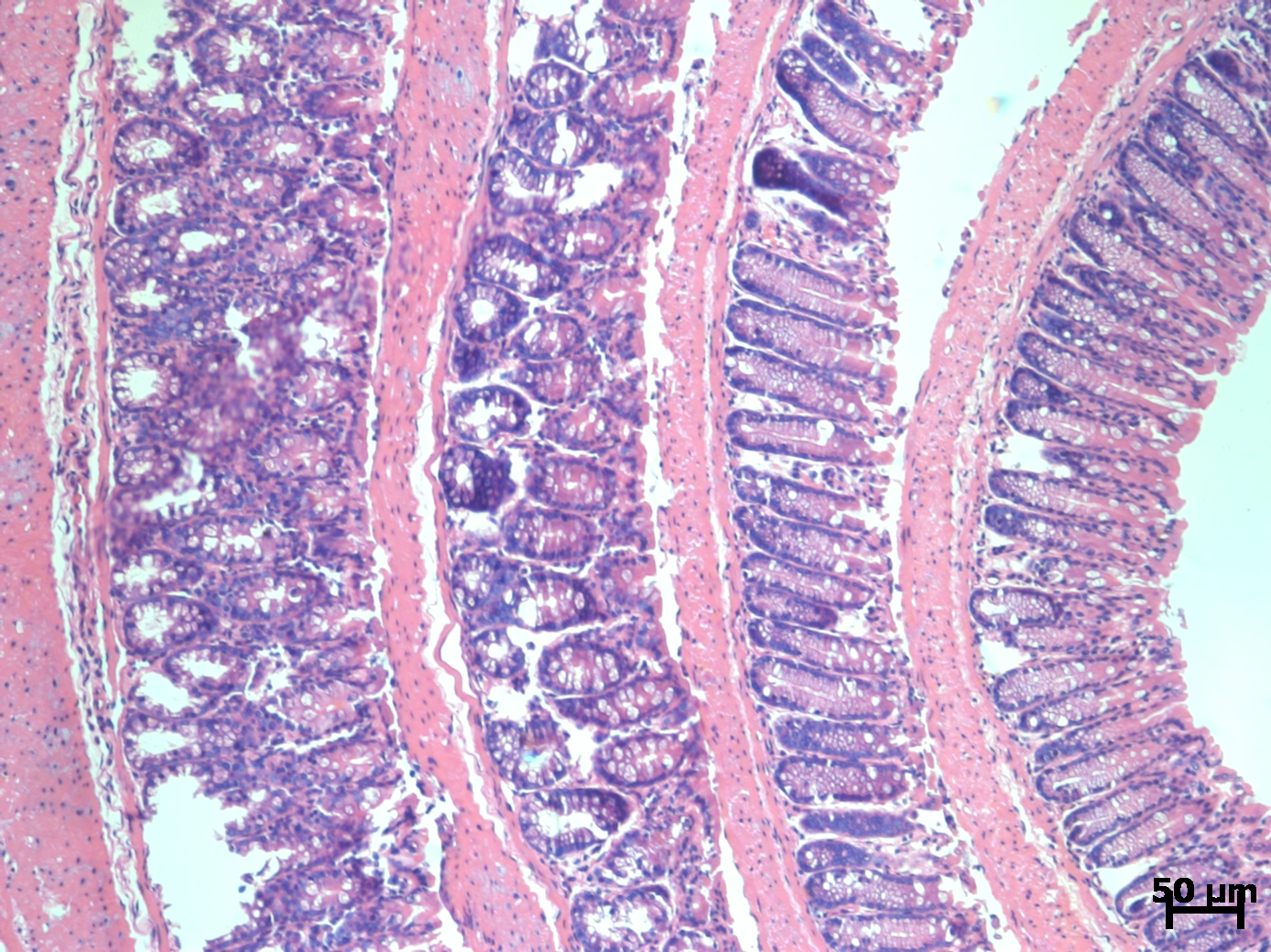

Supplement: Supplementary file 3 — Source data Fig. 1 [file 44318_2024_182_MOESM3_ESM.zip › EMBOJ-2024-117451R1_SourceDataFor Figure 1/Figure 1I/Control.tif]

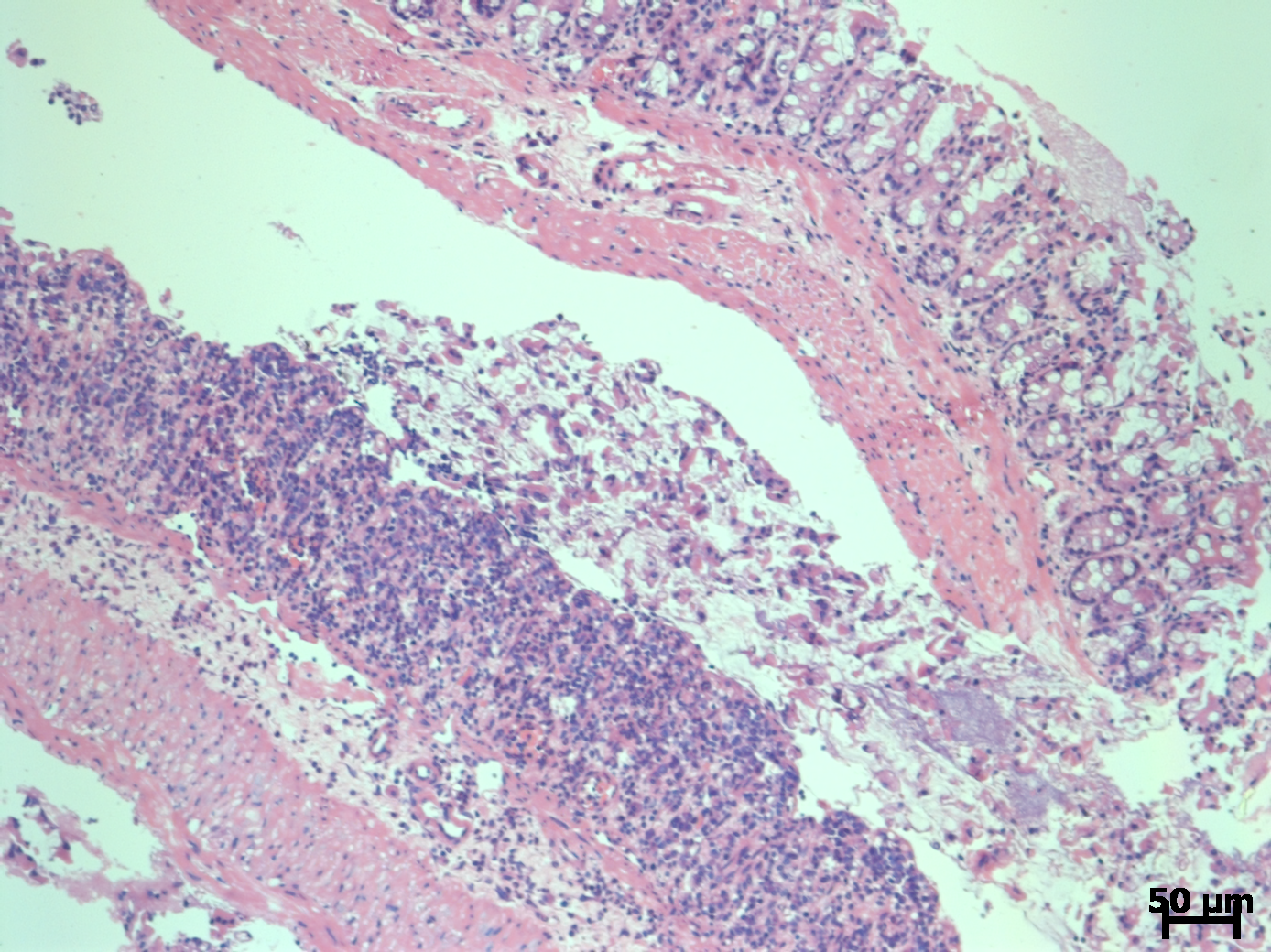

Supplement: Supplementary file 3 — Source data Fig. 1 [file 44318_2024_182_MOESM3_ESM.zip › EMBOJ-2024-117451R1_SourceDataFor Figure 1/Figure 1I/Control+DSS.tif]

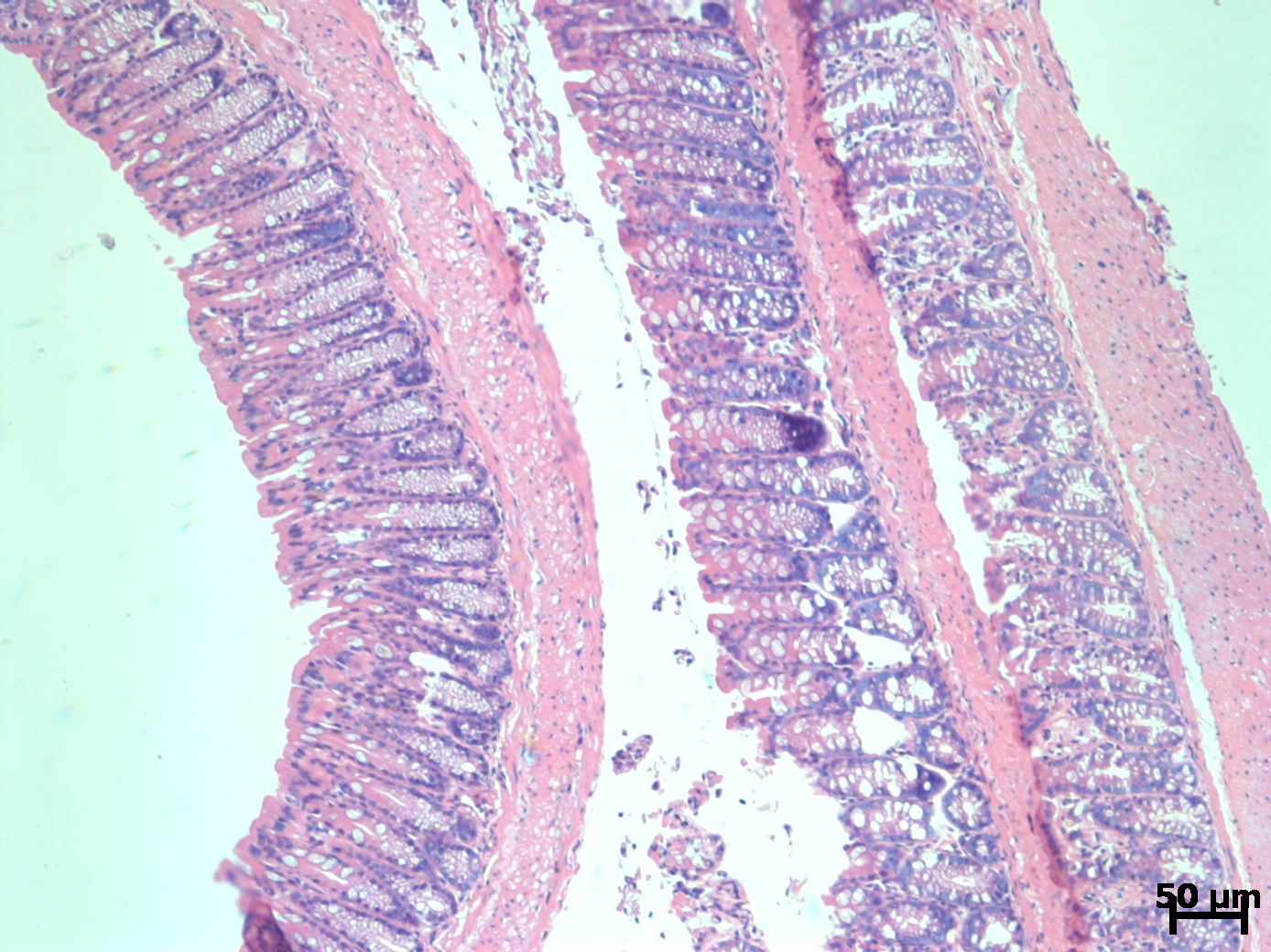

Supplement: Supplementary file 3 — Source data Fig. 1 [file 44318_2024_182_MOESM3_ESM.zip › EMBOJ-2024-117451R1_SourceDataFor Figure 1/Figure 1I/KO.tif]

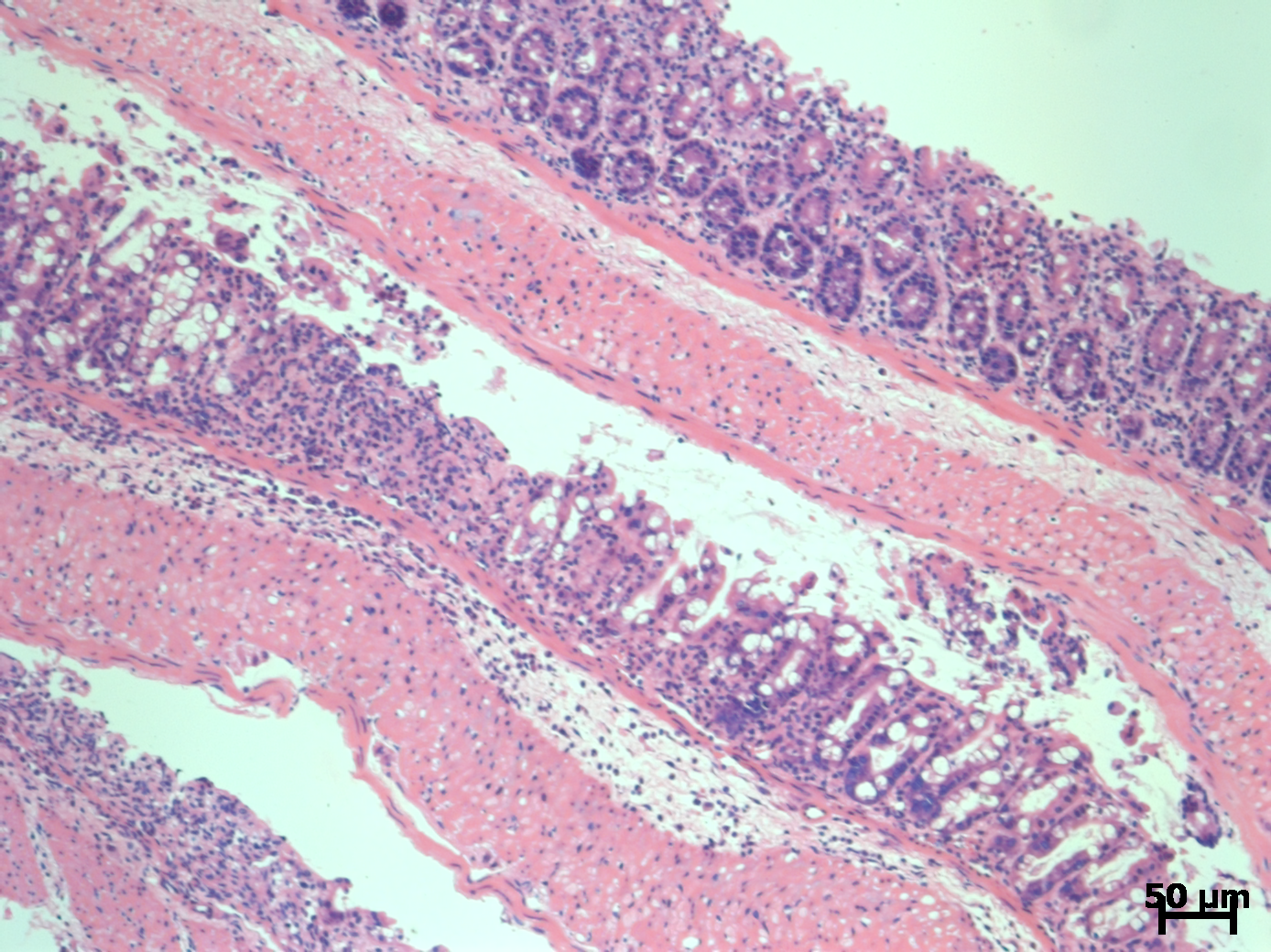

Supplement: Supplementary file 3 — Source data Fig. 1 [file 44318_2024_182_MOESM3_ESM.zip › EMBOJ-2024-117451R1_SourceDataFor Figure 1/Figure 1I/KO+DSS.tif]

Figure2 – Source Data

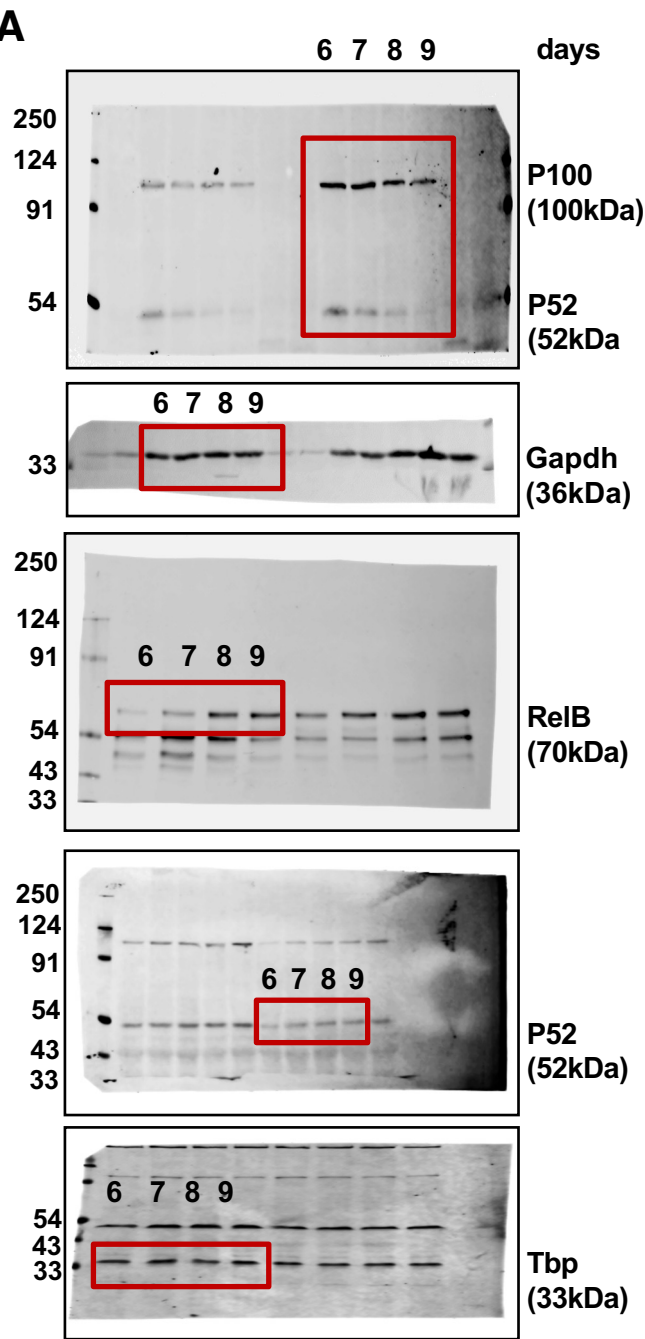

Supplement: Supplementary file 4 — Source data Fig. 2 [file 44318_2024_182_MOESM4_ESM.zip › EMBOJ-2024-117451R1_SourceDataFor Figure 2/Figure 2A.pdf]

Figure2 – Source Data

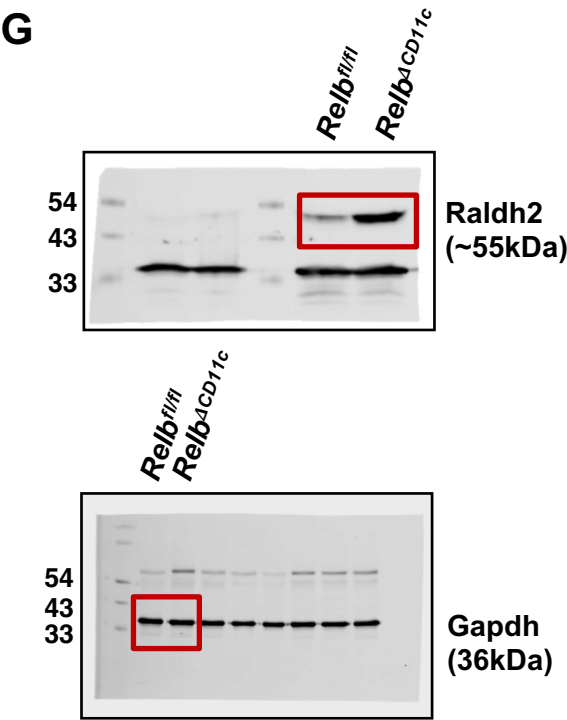

Supplement: Supplementary file 4 — Source data Fig. 2 [file 44318_2024_182_MOESM4_ESM.zip › EMBOJ-2024-117451R1_SourceDataFor Figure 2/Figure 2G/Figure 2G.pdf]

Figure5A – Source Data

*Relb*<sup>fl/fl</sup> - 1  
*Relb*<sup>ΔCD11c</sup> - 2

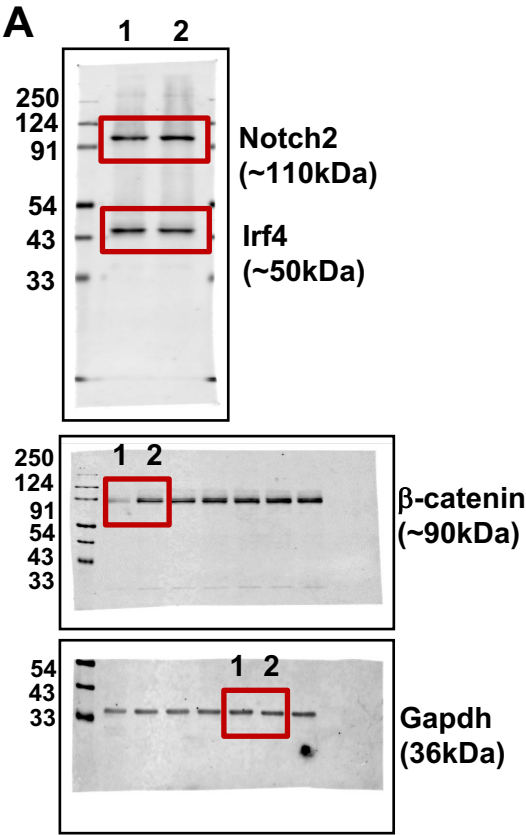

Supplement: Supplementary file 7 — Source data Fig. 5 [file 44318_2024_182_MOESM7_ESM.zip › EMBOJ-2024-117451R1_SourceDataFor Figure 5/Figure 5A.pdf]

Figure5C – Source Data

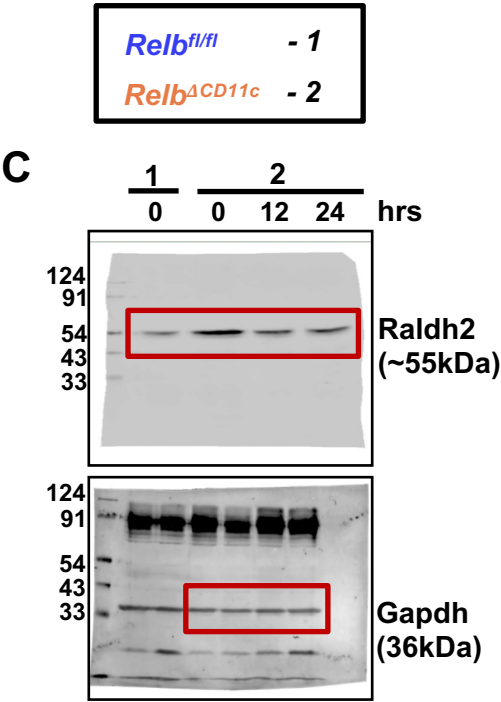

Supplement: Supplementary file 7 — Source data Fig. 5 [file 44318_2024_182_MOESM7_ESM.zip › EMBOJ-2024-117451R1_SourceDataFor Figure 5/Figure 5C.pdf]

Figure5D – Source Data

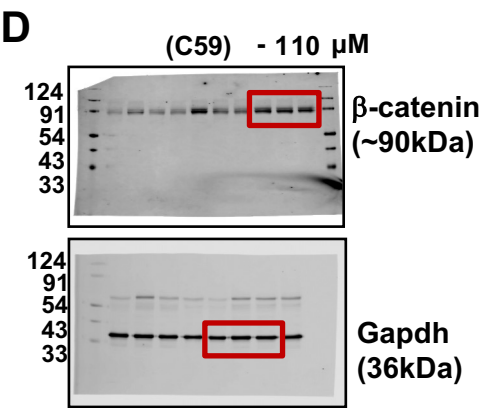

Supplement: Supplementary file 7 — Source data Fig. 5 [file 44318_2024_182_MOESM7_ESM.zip › EMBOJ-2024-117451R1_SourceDataFor Figure 5/Figure 5D.pdf]

Figure5H – Source Data

|                              |     |
|------------------------------|-----|
| <i>Relb<sup>fl/fl</sup></i>  | - 1 |
| <i>Relb<sup>ΔCD11c</sup></i> | - 2 |

H

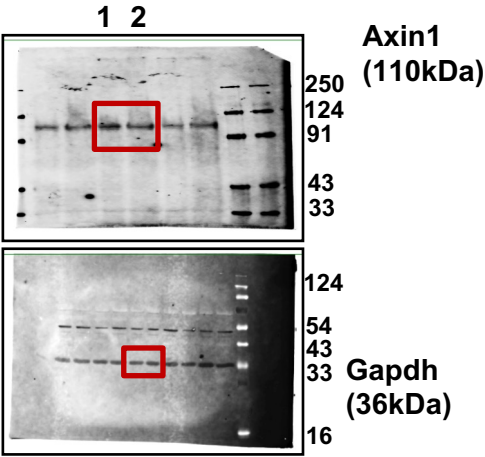

Supplement: Supplementary file 7 — Source data Fig. 5 [file 44318_2024_182_MOESM7_ESM.zip › EMBOJ-2024-117451R1_SourceDataFor Figure 5/Figure 5H/Figure 5H.pdf]

Figure5I – Source Data

*Relb*<sup>fl/fl</sup>

- 1

*Relb*<sup>ACD11c</sup>

- 2

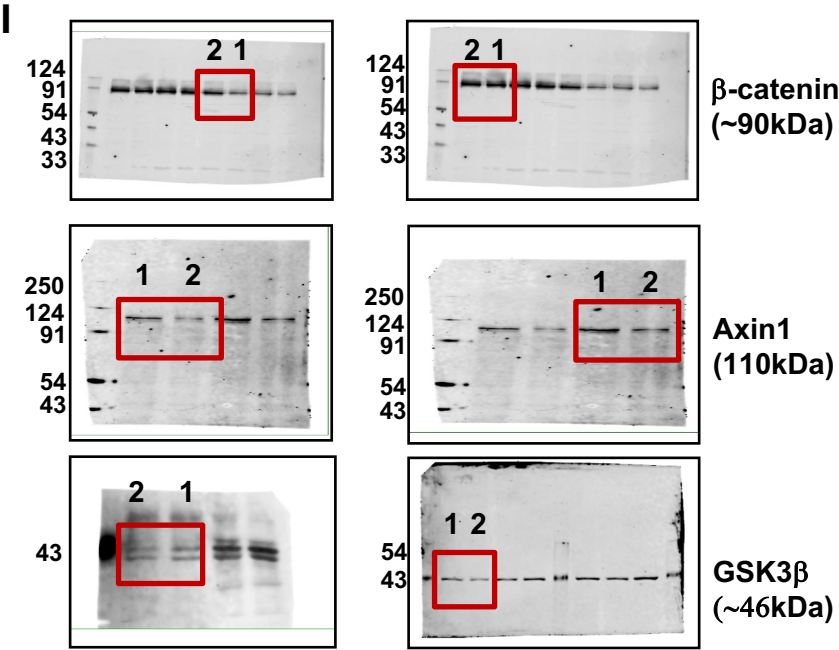

Supplement: Supplementary file 7 — Source data Fig. 5 [file 44318_2024_182_MOESM7_ESM.zip › EMBOJ-2024-117451R1_SourceDataFor Figure 5/Figure 5I.pdf]
