# Supplementary material for: Non-canonical NF-κB signaling limits the tolerogenic β-catenin-Raldh2 axis in gut dendritic cells to exacerbate intestinal pathologies
Source: EMBO J. 2024 Jul 25;43(18):3895–915. doi: 10.1038/s44318-024-00182-6 (PMC11405688; doi:10.1038/s44318-024-00182-6)

Figure5B – Source Data

$Relb^{fl/fl}$	*- 1*	*$Nfkb2^{fl/fl}$*	*- 3*
$Relb^{\Delta CD11c}$	*- 2*	*$Nfkb2^{\Delta CD11c}$*	*- 4*

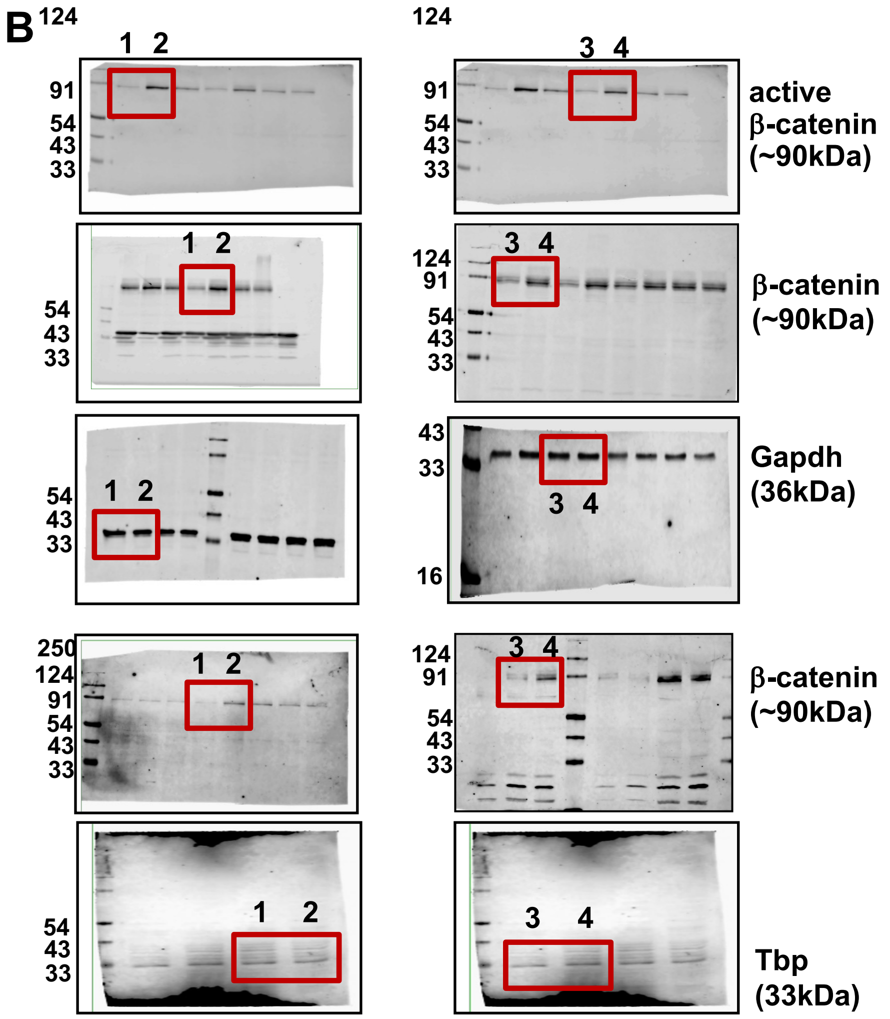

Supplement: Supplementary file 7 — Source data Fig. 5 [file 44318_2024_182_MOESM7_ESM.zip › EMBOJ-2024-117451R1_SourceDataFor Figure 5/Figure 5B/Figure 5B.pdf]
